# Are Discrepancies in the Amount of Radiation Delivered to Target Neoplastic Cells and Teeth in Cases of Head and Neck Cancer Significant Considerations in Laboratory Research?

**DOI:** 10.1055/s-0044-1785189

**Published:** 2024-05-28

**Authors:** Lucas da Fonseca Roberti Garcia, Lívia Ribeiro

**Affiliations:** 1 Endodontics Division, Department of Dentistry, Health Sciences Center, Federal University of Santa Catarina, Florianópolis, SC, Brazil

Each year, 2.4 million new cases of head and neck cancer (HNC) are reported, resulting in 1.3 million deaths. 1 HNC is a comprehensive term for neoplasms that develop in areas such as the mouth, tongue, soft and hard palate, buccal mucosa, tonsils, pharynx, larynx, esophagus, thyroid, paranasal sinuses, nasal cavities, and salivary glands. 1 The therapeutic approach for HNC often involves a combination of surgery and radiotherapy, or alternatively, multimodal therapy combining chemotherapy with other modalities of treatment. 2 Radiotherapy uses ionizing radiation as a therapeutic agent. 3 High-energy radiation acts directly on the breaking of deoxyribonucleic acid (DNA) chains of neoplastic cells, or indirectly, on the production of free radicals and hydrogen peroxide, leading to cellular apoptosis. 4

The assessment of an oncological patient who will undergo radiotherapy is conducted by a medical radio-oncologist, who, in collaboration with the medical physicist, develops the radiotherapeutic plan. 1 The plan includes the precise identification of the area to be irradiated (target tissues), the protection of regions to be preserved from irradiation, the determination of the radiation dose, and the establishment of the required number of radiotherapy sessions. 1 The radiation dosage used throughout the treatment of HNC ranges from 55 to 70 Gy. 5 The radiation primarily targets the center of the tumor mass. 5 The point that deserves special attention is the exact amount of radiation delivered to the anatomical structures close to the tumor area, especially the maxilla, mandible, salivary glands, and teeth. 6 Among the clinical variables that may influence the radiation dose delivered to these structures are the extent and location of the tumor. 7

The survival rate of patients with HNC has increased in recent decades, 8 justifying research that evaluates the effect of radiation on dental structures. 3 4 9 10 11 However, due to the inherent difficulties in conducting clinical research on oncological patients undergoing radiotherapy, most studies are conducted in a laboratory setting, 3 4 9 11 aiming to closely mimic clinical situations as much as possible.

The analysis of dosimetric maps demonstrates that the radiation delivered in the radiotherapeutic treatment of different types of HNC results in varying radiation doses delivered to the maxilla, mandible, and teeth. 7 12 Polce et al 12 reported that a case of nasopharyngeal cancer treated with 60 Gy delivered only 30 Gy to the lower premolar region. Conversely, a larger lesion in the oropharyngeal region delivered 63 Gy to the lower premolars. 12 Therefore, the amount of radiation delivered to the teeth is not always the same as that intended for the target neoplastic cells. Laboratory studies using radiation doses ranging from 60 3 4 to 70 Gy 10 11 may overestimate the damage presented by dental structures.

Walker et al 13 clinically demonstrated a direct relationship between the increase in radiation dose and damage to dental structures. Radiation doses below 30 Gy cause minimal dental damage, which tends to increase with doses between 30 and 60 Gy, reaching a critical threshold with doses above 60 Gy. 13 Velo et al 9 observed in their *in vitro* study that higher doses result in greater damage to teeth.

Combining this information, we may pose some questions. Campi et al 4 used in their laboratory study 20 homologous lower premolars. The authors assessed chemical changes in dentin irradiated with 60 Gy. The irradiation protocol was similar to that used for HNC patients undergoing radiotherapy with fractionated doses of 2 Gy, for 5 consecutive days, with 30 cycles over 6 weeks. However, as described above, depending on numerous factors, such as location, extension, and tumor type, the dose delivered to lower premolars may range. The authors 4 concluded that radiotherapy induced changes in the concentration of phosphate, carbonate, and peaks of amide III in root dentin. However, does this result reflect the actual clinical radiotherapeutic damage, or does it present the chemical variation of dentin subjected to 60 Gy of irradiation?

Reed et al, 11 also in a laboratory study, used sections of seven noncarious third molars to assess the enamel structure, the mechanical properties, and the chemical composition of dentin before and after the application of a total dose of 70 Gy. In this context, according to the study by Polce et al, 12 70 Gy seems to be a high and specific estimate for certain types of cancer. Polce et al 12 demonstrated that for the treatment of a large nasopharyngeal cancer, the lower third molars may receive an average of 66 Gy, whereas, for an early-stage glottic cancer, the average radiation dose was 0.25 Gy. Reed et al 11 did not limit the research to lower third molars; however, it is possible to understand that clinically, patients receive different doses of radiation, which is intrinsically related to specific factors of each type of HNC.

Recent studies from our research group have also followed the laboratory protocols used so far for teeth irradiation. Our findings indicate a significant decrease in the bond strength between fiberglass post/resin cement and irradiated root dentin, 14 as well as a reduction in the fracture resistance of simulated immature permanent teeth. 15 From this discussion, it is possible to suggest that, despite their importance in building scientific knowledge regarding the effects of radiotherapy on dental structures, laboratory studies conducted so far may not be simulating the ideal clinical scenario of radiotherapeutic treatment. Such studies have been and are fundamental for understanding the deleterious effects of radiation on dental structures. However, it is essential to continue considering methodological improvements so that such complex phenomena may be understood more clearly, always with the well-being of oncological patients in mind. The introduction of concepts like laboratory-on-chip technology, organoids, or employing machine learning models could significantly extend research endeavors on the effects of radiation on dental structures in cancer-related studies. Integrating these ideas could catalyze a broader discussion and pave the way for more extensive investigations.

It is important to emphasize that the purpose of this editorial is not to criticize laboratory studies simulating irradiated teeth, but rather to raise questions to improve the tests. Despite the inherent limitations of *in vitro* studies, the design of research should follow reliable perspectives to avoid overestimating their results. Doses ranging from 55 to 70 Gy are the averages administered to target neoplastic cells, not necessarily the doses that reach dental structures. Therefore, further research should be based on dosimetric maps, considering that the results should be restricted to predefined conditions, such as tumor type and location, and should not be extrapolated to all types of HNC.
